# A Thermodynamic Model of Microtubule Assembly and Disassembly

**DOI:** 10.1371/journal.pone.0006378

**Published:** 2009-08-11

**Authors:** Bernard M. A. G. Piette, Junli Liu, Kasper Peeters, Andrei Smertenko, Timothy Hawkins, Michael Deeks, Roy Quinlan, Wojciech J. Zakrzewski, Patrick J. Hussey

**Affiliations:** 1 Biophysical Sciences Institute, University of Durham, Durham, United Kingdom; 2 Department of Mathematical Sciences, University of Durham, Durham, United Kingdom; 3 School of Biological and Biomedical Sciences, University of Durham, Durham, United Kingdom; University of Waterloo, Canada

## Abstract

Microtubules are self-assembling polymers whose dynamics are essential for the normal function of cellular processes including chromosome separation and cytokinesis. Therefore understanding what factors effect microtubule growth is fundamental to our understanding of the control of microtubule based processes. An important factor that determines the status of a microtubule, whether it is growing or shrinking, is the length of the GTP tubulin microtubule cap. Here, we derive a Monte Carlo model of the assembly and disassembly of microtubules. We use thermodynamic laws to reduce the number of parameters of our model and, in particular, we take into account the contribution of water to the entropy of the system. We fit all parameters of the model from published experimental data using the GTP tubulin dimer attachment rate and the lateral and longitudinal binding energies of GTP and GDP tubulin dimers at both ends. Also we calculate and incorporate the GTP hydrolysis rate. We have applied our model and can mimic published experimental data, which formerly suggested a single layer GTP tubulin dimer microtubule cap, to show that these data demonstrate that the GTP cap can fluctuate and can be several microns long.

## Introduction

Microtubules are dynamic filaments that perform essential functions in eukaryotic cells including nuclear and cell division and intracellular transport. A microtubule is a cylindrical assembly of tubulin dimers which are composed of *α* and *β*-tubulin subunits. These dimers associate head to tail to form protofilaments and usually 13 protofilaments associate laterally to form the wall of the microtubule. The protofilaments are slightly shifted with respect to each other and after one full turn of the microtubule there is a total shift of 1.5 dimers at the seam, the join of the first and thirteenth protofilament in the microtubule[1].

The tubulin dimer has two GTP binding sites a non-exchangeable GTP site at the interface of the *α* and *β* subunits and an exchangeable site between the dimers in a protofilament. At the exchangeable site GTP hydrolysis occurs 250 times faster when the GTP tubulin dimer is bound within the microtubule compared to when it is in free solution [2]. GTP-tubulin dimers have a slightly altered conformation compared to GDP-tubulin dimers and as a result only the former can assemble into microtubules. When hydrolysis of GTP takes place within the microtubule it is the neighbouring dimer interactions that hold the GDP dimers in place [3].

The asymmetry of the tubulin dimer is translated into the microtubule as it assembles and the exposed *β* tubulin end is called the plus end and the other end the minus end. Each of the plus and minus ends have different properties in respect of structure and growth [4]. When sufficient free GTP tubulin dimer is present microtubules will grow and the result is a cap of GTP tubulin dimer on the microtubule. As the growth rate slows the cap is lost as the GTP is hydrolysed to GDP in the tubulin dimer. As the binding energy of GDP-tubulin dimer is lower than that of GTP-tubulin the microtubule undergoes rapid shortening. This self-assembly and disassembly of microtubules is known as dynamic instability [5].

Modelling microtubule dynamics is giving further insight into the manner of microtubule assembly and disassembly. So far the best approach to model microtubule dynamics has been to use Monte Carlo simulations which were first performed by Chen and Hill [6], [7] initially for a single protofilament then progressing to a 13 protofilament microtubule. This model had 17 parameters some of which were chosen so as to reproduce the experimental values of Mitchison and Kirschner [5]. A few years later, Bayley and colleagues [8] proposed a similar model, except that GTP tubulin dimers were assumed to hydrolyse spontaneously once embedded inside the microtubule. Such models lead to a GTP tubulin cap that is only one heterodimer long. However, Van Buren et al [9] have introduced a model where the number of parameters was reduced to 4 parameters per microtubule end by relating the tubulin attachment and detachment rates to the binding energy of tubulin heterodimers.

In this paper we derive a thermodynamic model for microtubule dynamics and use this model to perform Monte Carlo simulation where we include the contribution of water to the entropy of the system and we fit all the parameters of our model to published experimental data including the hydrolysis rate of GTP. We consider both the + and the − ends. Moreover, using our model we have reinterpreted existing experimental data [10] that were used to predict a short GTP cap to show that the cap can be several microns long and dependent on the concentration of free GTP-tubulin dimer, which is now consistent with recent observations [11].

## Results

### Theoretical model: thermodynamics

**Figure 1:**
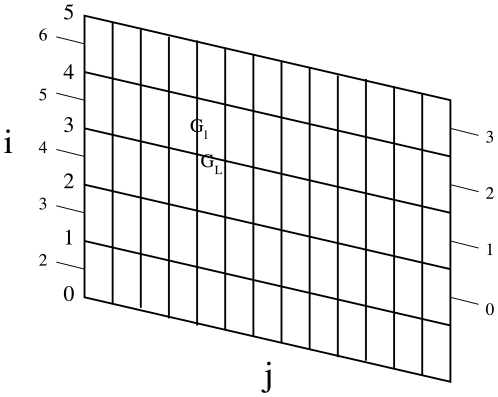
Unfolded microtubule lattice. Two dimensional representation of the unfolded lattice for a 13 protofilament microtubule configuration. The lateral displacement between protofilaments results in a seam where the monomers are attached to a monomer of the other type, as shown by the labeled lines on the sides of the figures.

**Figure 2:**
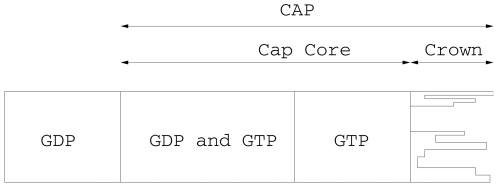
Detailed structure of the microtubule cap. to 7 cm.

In this paper, we consider the microtubule as a lattice with a 1.5 dimer shift at the seam (Fig. 1). We also view the cap as having two components: a crown consisting of incomplete protofilaments and the core that forms the body of the complete microtubule and includes GTP tubulin dimers (Fig. 2).

The polymerisation of microtubules involves 3 types of reactions. First of all, GTP-tubulin dimers, which we denote by 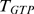, can attach or detach from the tip of the microtubule. GDP-tubulin dimers, 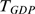, on the other hand, can only detach from the microtubule tip. Then, 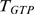 inside the microtubule can hydrolyse into 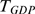.

To describe the polymerisation of microtubules one must consider all the possible configurations that a microtubule can take. Each microtubule is characterised by the length of each protofilament as well as the type of every dimer in each of these protofilaments. The number of possible microtubule configurations, which we call 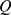, is thus extremely large and instead of using a sophisticated labeling system to describe each of these configurations, we have decided to label them formally using a single index *J* that takes 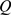 different values. As we will not need to consider the details of each configuration, this formal parametrisation has the advantage of being both simple and sufficient for what we want to do.

We then denote by 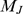 a microtubule in the configuration *J*. Each time a tubulin dimer detaches from or attaches itself to a microtubule, or each time a GTP-tubulin dimer hydrolyses to a GDP-tubulin, the configuration of the microtubule changes.

The polymerisation of microtubules involves 3 types of reactions. First of all, GTP-tubulin dimers, which we denote by 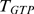, can attach or detach from the tip of the microtubule. GDP-tubulin dimers, 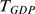, on the other hand, can only detach from the microtubule tip. Then, 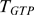 inside the microtubule can hydrolyse into 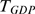. These three reactions, which all have their own reaction rates described later, can be summarised as follows: 
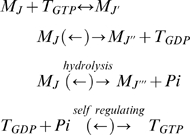
(1)where 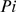 is an inorganic phosphate. We have added here, for completeness, the self regulating process transforming GDP-tubulin into GTP-tubulin. Note also that we have enclosed within parentheses the back reaction arrows for the last 3 equations in (1) because, while their reaction rates are very small, they must still be considered for a full thermodynamic description of the system. In each of these equations, the indices *J*, 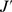, 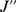 and 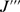 are related to each other but we still have a very large number of simultaneous reactions involving different concentrations of various microtubule configurations. Equation (1) is a formal system of equation corresponding to a very large number of chemical equations, one for every combination of related indices.

The last equation in (1) symbolises the self regulatory process that controls the GTP-tubulin concentration in the cell. For *ie* in vitro experiments, the number of microtubules is usually small enough that the GTP-tubulin concentration remains constant. For both types of experiments we will thus assume, in what follows, that the GTP-tubulin concentration is constant.

It is important to realise that each microtubule can transform into many other microtubule configurations and that each microtubule can also be obtained from many other microtubule configurations. A specific microtubule configuration 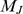 can occur on both sides of the first 3 reactions in (1) for a small set of explicit values of *J*, 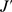, 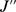 and 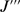. Equilibrium for (1) can then be achieved because the microtubules decaying, say by hydrolysis, are recreated through a combinations of all 4 equations.

We now consider a volume *V* containing 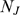 moles of microtubules in configuration *J*, 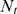 moles of free GTP-tubulin dimers, 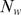 moles of water, 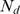 moles of free GDP-tubulin dimers and 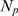 moles of Pi. A priory, we thus have 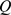 different types of microtubules, each with their own concentration 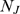. Moreover, we know that in physiological conditions, 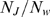, 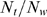, 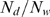 and 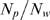 are always very small.

The Gibbs' energy of the microtubule solutions is given by [12]

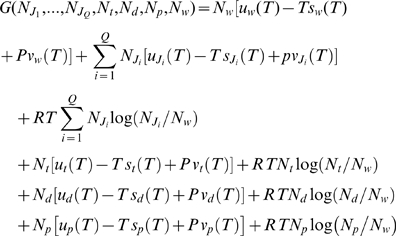
(2)where 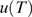, 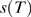 and 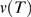 are, respectively, the energy, the entropy and the volume of one mole of the different constituents and each of these quantities depends on the temperature *T*. As a matter of fact in (2), for each solute, *i.e.* not for water, we have 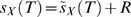, where 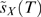 is the entropy of the corresponding solute while *R* is the entropy gained by the solvent, water, as the solute is added to the solution. 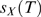 and 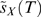 are thus two valid expressions of entropy but which have different physical origins. The use of one rather than the other will not affect our results as such but it will modify the interpretation of some of the quantities discussed later.

For a perfect solution, the energies 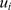 and the entropies 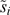 are, respectively, the internal energies and internal entropies of each substance.

In (2), 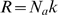 where 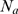 is Avogadro's number and *k* is the Boltzmann constant.

During the association of one tubulin dimer to a microtubule 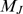 to produce a microtubule 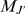, as in the first reaction in (1), the Gibbs' energy changes and this variation can be easily computed as
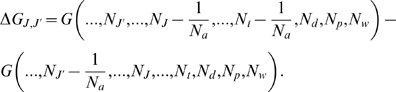
(3)


Note that the variations of Gibbs' energy during the dissociation, as in the first two reactions in (1), are also given by (3), after changing the signs on the right hand side and, for the detachment of GDP-tubulin, exchanging 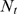 and 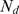.

We have decided to compute all the quantities normalised to single molecules rather than 1 mole because we want to derive a model based on a Monte Carlo simulation of the chemical reaction which deals with only one molecule at a time.

Using the fact that 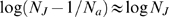 we have
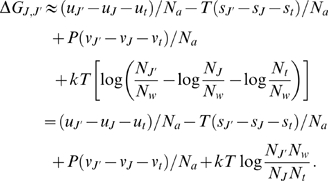
(4)


Note that an equation like (4) can also be used, up to a sign, for the detachment of any type of tubulin dimer. A similar expression can be used to compute the variation of the Gibbs' energy during the hydrolysis, but as we will not need it we do not present it here.

Denoting, respectively, the energy, entropy and volume per molecule as 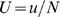, 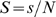 and 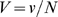, we define the variation of energy, entropy and volume during the reaction as:
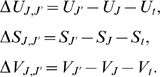
(5)


Notice that the variation of energy 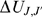 is the energy necessary to detach a single tubulin dimer from the microtubule in configuration *J* to obtain one in configuration 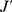 in the considered solution. In an ideal solution, this is just the binding energy of the molecules while in a non-ideal case, 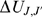 will also include the variation of all electrostatic/chemical interactions and of the average configuration changes of all the tubulin dimers, bound or not, and of the solution itself. This could, in theory, be computed from the full Hamiltonian of the system which includes all the interactions ( *ie* chemical, or electrostatic) between the different substances present in the solution. A given 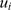 in (2) will thus be the sum of the internal energy and of half the interaction energy with the other constituents. By symmetry, each interaction term will occur twice, hence the need to half them. One should also take into account the presence of other solutes, such as ions or proteins , always present in experimental assays or in *in vivo* solutions. Such solutes do not play a direct role in the dynamics of the microtubule, but they can affect indirectly the binding energies of the tubulin dimers and hence, as we will see, have an indirect impact on the microtubule dynamics.

One could, ideally, evaluate these variations of energies by solving the Schrödinger equation describing the system, but in practice this is impossible given the number of electrons involved. An alternative would be to use semi-classical methods to approximate the solutions of the Schrödinger equation. Then, the interaction between molecules will be described by a collection of tailored chemical and electrostatic potentials describing the interactions between the tubulin dimers, water molecules and the other substances present in the solution [13].

In practice, the cytoplasmic solution of microtubule and tubulin is not an ideal solution in the thermodynamic sense as there are non zero forces between all the molecules involved. 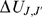 thus contain contributions which, in principle, could be computed ab initio, but which are usually described by the activity parameters for the reaction. In practice we will fit the values of 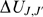 to experimental data without attempting to derive them.

Note also that
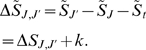
(6)


### Microtubule at Equilibrium

When microtubules are studied experimentally either in *ie* in vitro or *in vivo* experiments, their dynamics is usually not at thermal equilibrium. Thermal equilibrium is reached when a solution has a very large number of microtubules polymerising and depolymerising according to (1) and where the concentration of all the solutes remain, on average, constant. As we will see below, this critical concentration is very special and it will allow us to link together the Gibbs' energy, computed above, to the attachment and detachment rates of tubulin dimers and to the value of the critical concentration.

The thermodynamic equilibrium is characterised by the condition 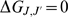 and the chemical potential 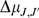 is defined as
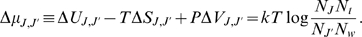
(7)


To evaluate this variation of the chemical potential, we assume that the entropy of a microtubule composed of 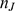 tubulin dimer is 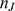 times the entropy of a single tubulin dimer: 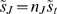. This assumes that the tubulin dimers have some vibrational degrees of freedom when they are attached to a microtubule. As a direct consequence, the entropy does not change during the binding process and 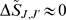. This in turn implies that, for a perfect solution 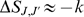. If our approximation were incorrect, then 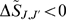. 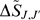 would also be non-zero for a non perfect solution, but for the sake of simplicity, we will take 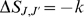 at this stage and we will show later that, if 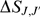 were different, the model, as it stands, would not be affected. Only the derived value of the longitudinal binding energy of a tubulin dimer would have to be changed.

In a liquid solution we have 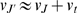 which implies that the volume of the system does not change significantly during the binding process either and 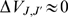. In our model, so far we have not taken into account the fact that the entropy of the system might change as a result of electrostatic interactions or because of some geometrical effects. For example, when a tubulin dimer detaches itself from the microtubule it exposes a larger area on which water molecules can cluster themselves on the protein. We would expect this to decrease the entropy.

We will assume that such entropy effects are small, but if they were not, they could be added to 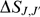 which would then be non zero. Similarly, because of the electrostatic interactions between the dimers and the solution, 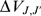 might not be small enough to be negligible. In either case, at constant temperature and pressure, non zero factors for 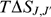 and 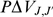 would just be constant contributions to 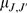 which would not affect the modeling results but, as we shall see later, would alter the interpretation of the binding energy.

Before we proceed further, we rewrite (7) so that it expresses the relative number of moles of the different microtubule types as well as GTP-tubulin *at equilibrium* as a function of the tubulin dimers binding energies:
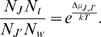
(8)


We should stress that this relation is only valid at the critical concentration, which corresponds to the thermodynamical equilibrium of the system, and where, moreover, the hydrolysis rate is small compared to the polymerisation and depolymerisation rates. Note also that (8) is dimensionally balanced.

### Microtubule (De)-Polymerisation Rates

Considering the same volume *V* as above, we must now analyse the rate at which the microtubule (de-)polymerises. First of all, we define 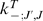 and 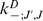 as the dissociation rates per microtubule of, respectively, a GTP-tubulin or GDP-tubulin dimer for a microtubule going from state 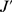 to *J*. 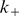 is then defined as the first order rate at which a microtubule polymerises to another state at a given concentration of free GTP-tubulin. GDP-tubulin does not polymerise, so 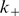 for GDP-tubulin is zero. As the mean free path of a tubulin dimer in a water solution at body temperature is very short when compared to its size, we can assume that 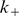 for GTP-tubulin does not depend on the the microtubule state. In other words, we assume that there are no geometrical factors such as there would be if we considered a gas. We also define 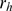 as the hydrolysis rate of GTP-tubulin into GDP-tubulin inside the microtubule and 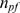 as the number of proto-filaments.

The rate of change of 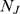 is given as the sum of all the transitions creating a microtubule in state *J* less the sum of all the rates of transitions from which state 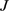
can decay. For the sake of convenience, we define the following sets of microtubule states:

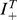
 : the set of 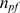 states which can be obtained from state *J* by adding a GTP-tubulin dimer.

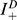
 : the set of 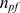 states which can be obtained from state *J* by adding a GDP-tubulin dimer.

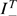
 : the set of states which can be transformed into the state *J* by removing one GTP-tubulin dimer.

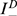
 : the set of states which can be transformed into the state *J* by removing one GDP-tubulin dimer.

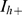
 : the set of states from which the state *J* can be obtained through the hydrolysis of one GTP-tubulin dimer.

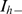
 : the set of states into which the state *J* can transform through the hydrolysis of one GTP-tubulin dimer.

Note that together, the two sets 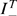 and 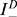 have a total of 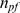 different states.

Using these definitions, the variation of 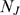, the number of microtubules in configuration *J*, is given by
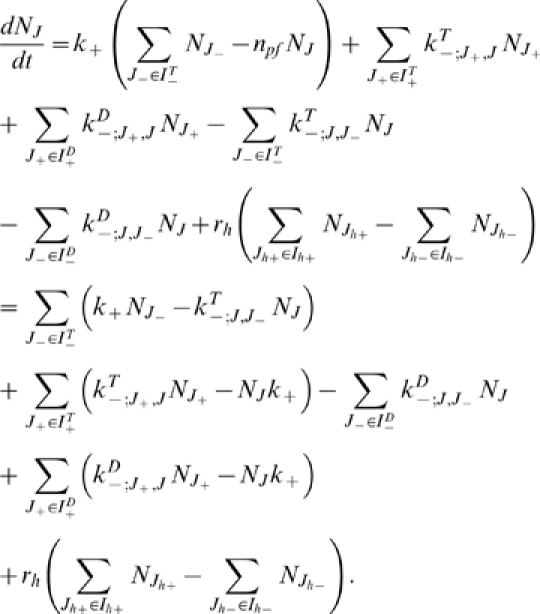
(9)


Eq. (9) states that the variation of the number of microtubules 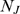 in a short time interval is equal to the number of microtubules 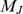 created by 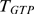 polymerisation or depolarisation, 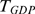 depolarisation or 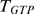 hydrolysis from microtubules in other configurations, reduced by the number of microtubules 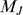 that are converted to other microtubule configurations also by polymerisation, depolarisation or hydrolysis.

At thermodynamic equilibrium, 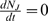 and, following [9], we can use that special configuration to determine a relation between 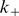, 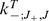 and 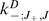. In the case of a pure GTP-tubulin microtubule when 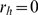, it is straightforward to show that
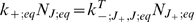
(10)is a solution of (9), where we have introduced the parameters 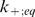, 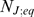 and 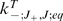 defined as the values of 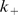, 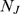 and 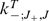, respectively, at thermodynamic equilibrium.

Eq. (10) states that if no hydrolysis takes place then microtubules are made entirely out of GTP-tubulin and so the polymerisation rates for 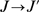 and depolymerisation rates for 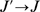 are the same.

When the hydrolysis rate, 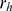, has a value comparable to the other dynamical rates, then finding a solution to (9) is very hard, and one cannot derive any expression like (10). This is because the ratios of 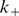 and 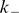 for different *J* and 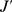 are not directly related to their relative concentrations anymore, but the rates and concentrations for different configurations are all interdependent.

If the hydrolysis rate is non-zero but small, (9) has several residual terms, but we can assume that they are all very small. Indeed, if 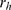 is small compared to 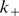 and 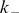, the GTP cap will be large, and there will be many states from which and into which the state *J* can hydrolyse. Moreover, on average, the number of these states will be very similar and they will have similar probabilities to be generated. We thus see that the 2 terms in the last sum in (9), proportional to 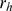, will mostly cancel each other out.

If the hydrolysis rate is small compared to 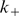, there will be very few GDP-tubulin dimers at the tip of the microtubule. This implies that there will be few microtubules in the configurations 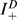 and 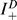 and that the sums over these configurations in (9) will be small. This will indeed be confirmed later by our simulations. In this case (9) is nearly correct and we can use it as a good approximation to the relation between 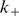 and 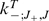 for the purpose of our study.

Note that in Eq. 9 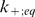, 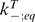, 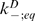 and 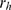 are all rates expressed in units of “per second per dimer” and they can thus all be compared with each other. To be able to use Eq. 10 we must have 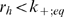, 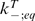 and 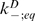. (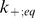 is also expressed in units of per second per dimer because it is defined as the attachment rate at the critical concentration of free GTP-tubulin).

To estimate 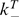 as a function of 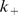, we ignore the hydrolysis rate and consider it as a small correction in the Monte Carlo simulation. This approximation will be justified if we show that, indeed, 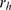 is small.

Within that approximation, we can use (8) and (10) and write
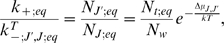
(11)which establishes a relation between the dissociation and association rates of microtubules and free tubulin *at the thermodynamical equilibrium*. As shown in (11) the ratio is related to the difference in energy of the 2 microtubule configurations and to their relative concentration *at the critical concentration*.

The dissociation rate of any dimer close to the tip of the crown can be computed using the expression
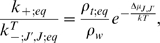
(12)where 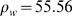 molar is the molar concentration of water and 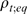 is the molar critical concentration of GTP-tubulin. We can thus use (12) to determine 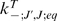 as a function of 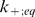 and of the binding energy difference which depends on *J* and 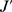.

It is also important to stress that the derivation of (12) is only valid at the equilibrium, *i.e.* only at the critical concentration 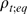. Moreover (12) assumes that 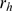 is small compared to 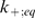 and 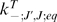. When the concentration 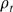 is different from 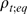, we do not have statistical equilibrium, but the only parameter that changes is 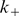 and we can write
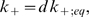
(13)where *d* is the relative tubulin concentration defined as the tubulin concentration normalised to the critical concentration:
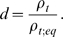
(14)


To perform a Monte Carlo simulation outside the equilibrium conditions, we use the fact 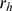 does not depend on 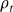 and that the dissociation parameters 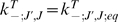 are the same as at the equilibrium and can thus be computed using (12). This is equivalent to the assumption that the detachment rate does not depend on the free tubulin concentration.

We must stress here once more that eq. (12), which relates the depolymerisation rates of the microtubules to their variation of internal energy, is only valid if the hydrolysis rate 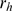 vanishes, or, as an approximation, if it is small compared to the first reaction at equilibrium. If 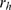 is too large, then there is no simple expression like (12). Instead, the depolymerisation rates and binding energies of all the possible states would depend on each other. Fortunately this turns out not to be the case.

### Monte Carlo Simulation

Our model involves a Monte Carlo approach to simulate the attachment and detachment of tubulin dimers to and from a microtubule. At any given time, several events can take place with different probabilities:

- A GTP-tubulin dimer can attach itself at the tip of the microtubule at the rate 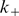 given by Eq. 13 .
- A GTP-tubulin dimer can hydrolyse into GDP-tubulin at the rate 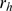. For the + end, as the exchangeable GTP of the GTP-tubulin dimers at the tip of any protofilament are not embedded inside the microtubule they hydrolyse very slowly and so we can put 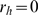 for these. All the other dimers hydrolyse at the same rate.
- Any dimer, GTP-tubulin or GDP-tubulin, can detach itself from the microtubule. The detachment rates 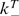 and 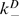 are obtained from Eq. 11 , 
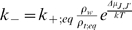
, where 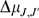 is the variation of chemical potential during the detachment, which depends on the microtubule configuration and on the position and the type of dimer.
- Strips of dimers are also allowed to detach together. In that case the variation of chemical potential 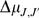 correspond to the total binding energy of the attached dimers. In practice, as 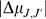 tends to be large, this occurs relatively rarely, except for dimers with no neighbours on one side.

As the detachment rate 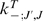 is independent of *d* it can be evaluated using Eq. 11 . To do this, we must determine 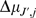. Note that to use Eq. 11 we must also know 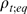 which has been determined experimentally by Walker et al. [14].

To evaluate 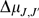 we use Eq. 7 and we observe that the binding energy of tubulin dimers can be split into the lateral and longitudinal binding energies which we denote, respectively, 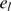 and 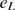 in units of 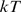. To follow the conventions of Van Buren et al [9], we define 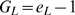, because 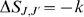, and 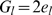 (see Fig. 1). Then we can write
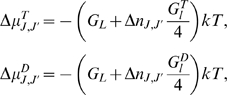
(15)where the superscript *T* and *D* refer, respectively, to GTP and GTP-tubulin and where 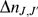 is the number of lateral monomer bonds that must be broken to go from configuration 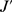 to configuration *J*. Note that by taking into account the entropy of water in Eq. 8 , the binding energy 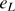 is 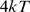 larger than the binding energy derived in [9] for an effective gas. Moreover, if we decide to modify the assumptions that we have made regarding the entropy 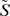 and assume 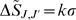 where 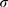 is a quantity that one would have to determine, then we would have 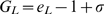. As 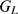 is the parameter that is fitted from experimental data, we see that our model is not affected by our assumptions, but that the value of the longitudinal binding energy 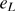, as a side product, is.

When several dimers detach themselves together from the tip of a protofilament, 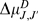 and 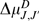 are still given by Eq. 15 and 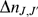 can be larger than 4. In practice, however, the detachment of several dimers when 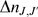 is large is a very rare event as such events are exponentially suppressed.

To evaluate 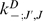 we also use Eq. 11, taking the same value for 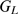 and 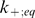 and for 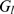 we take the value that fits the GDP-tubulin depolymerisation rate which we call 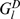. In doing so we exploit the fact that GDP-tubulin and GTP-tubulin are very similar dimers and we assume that the difference of binding energy comes from the lateral bounds; as GDP-tubulin is curved[15], it is less bound than GTP-tubulin.

To evaluate 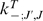 and 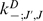 we must thus determine the three binding parameters 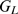, 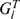 and 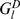. Ideally, one would like to derive them by performing an ab initio computation of the binding energies between tubulin dimers but this would be extremely difficult and, instead, we have determined their values using the microtubule dynamics experimental data[14].

As stated above, the rates 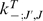, 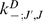, 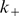 and 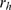 for the four types of events that takes place in the model are all expressed in the same units and can thus be used in a first reaction Monte Carlo simulations [16], [17] as follows. For any event with rate *k*, we computed the time 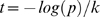 where *p* is a random number in the range 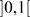 and we do this for the following events:

- For every GTP-dimer in the microtubule, we consider its hydrolysis at rate 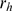 and its detachment together will all dimers attached longitudinally above it with rate 
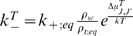
.
- For every GDP-dimer in the microtubule, we consider its detachment and its hydrolysis together will all dimers attached longitudinally above it with rate 
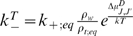
.
- For every tubulin dimer with no other dimer about it, *i.e.* tubulin dimers at the tip of their protofilament, we also consider the addition of a dimer to the same protofilament with rate 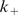.

From this large number of random times, we picked the shortest one, 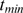, and selected the event to which it corresponds. We then implemented that event and increased the simulation time by 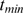, repeating this procedure for as long as required.

In summary, our model has 9 parameters: the relative free tubulin concentration *d*, the GTP hydrolysis rate 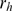 and the longitudinal free energy 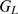, which have the same values for the + and the − end as well as the attachment rate 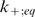 and the lateral binding energies 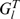 and 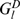 which have different values at the + and the − end.

### Parameter fitting with Experimental Data

To fit the values of the parameters of our model, we have used two sets of experimental data. The first set is made out of the parameters of the microtubule dynamics measured by Walker et al. [14], for both the + and − ends, and which are summarised by the expression
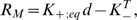
(16)where 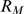 is the growing rate of the microtubules at relative concentration *d*, defined by Eq. 14 , while 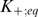 and 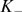 are the average microtubule attachment and detachment rates.

At the critical concentration, by definition, 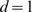 and 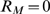 and so 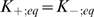. Walker et al. found that the critical concentration 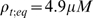 and that, when there were no catastrophes, 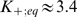 dimers/s/protofilament for the + end and 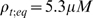 and 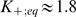 dimers/s/protofilament for the − end. In the catastrophe mode, the depolymerisation rate of GDP-tubulin 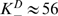 dimers/s/protofilament for the + end and 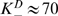 dimers/s/protofilament for the − end.

The second set of data comes from the measurement of the time delay before the onset of microtubule depolymerisation performed by Walker et al. [10]. In this experiment, microtubules were polymerised and the growing rates of microtubules measured. Then the solution was washed out by a solution free of tubulin and the time elapsed before the microtubule starts to depolymerise in a catastrophe was measured. The delay observed was roughly equal to 10 seconds, increasing slightly with the initial growing rate.

To ensure that our results are not affected by programming errors, the Monte Carlo simulations for our model were implemented and run totally independently by two of the authors. Their results matched perfectly.

**Figure 3:**
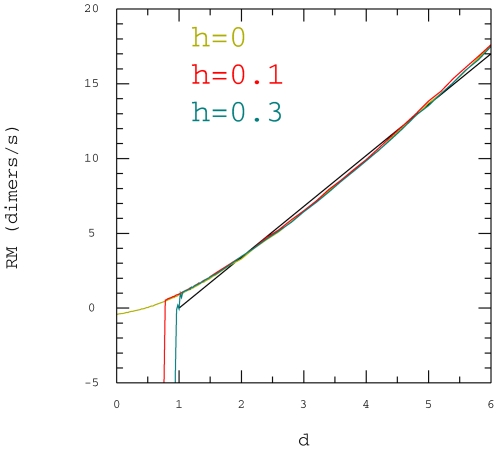
Growing rate of microtubule. Microtubule growing rate (dimer/s/protofilament) as a function of the relative concentration *d*, Eq. 14 , at the + end. Black line: experimental data from [14]. 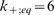, with (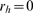, 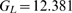, 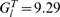), (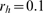, 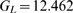, 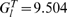, 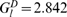), (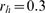, 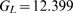, 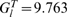, 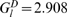).

Our first observation is that the growing rate of the microtubule is not linear as a function of the free tubulin concentration but that it is nearly linear in the range covered by the experiments in [14] and [10] (as seen in Fig. 3). This is why we took only 2 values for the concentration to fit the parameters.

To find values of 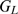 and 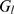 to reproduce the experimental data we had to take a 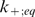 value larger than 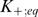. To decide which value of 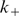 is best, we had to compare the growing rate of the microtubule as a function of the concentration and compare it to the experimental curve for Eq. 16 in [14].

**Figure 4:**
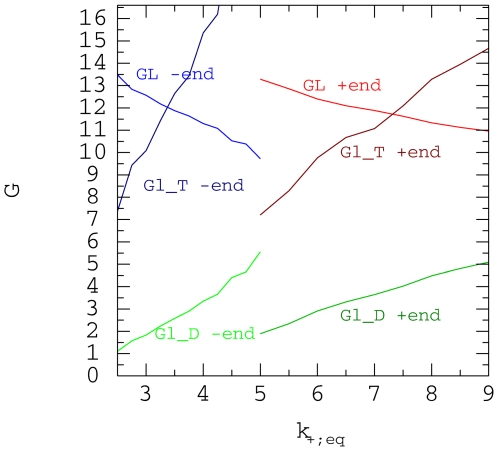
Binding energies. Binding energies 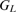, 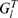 and 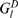 as a function of 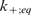 for the + and − end for 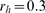.

**Figure 5:**
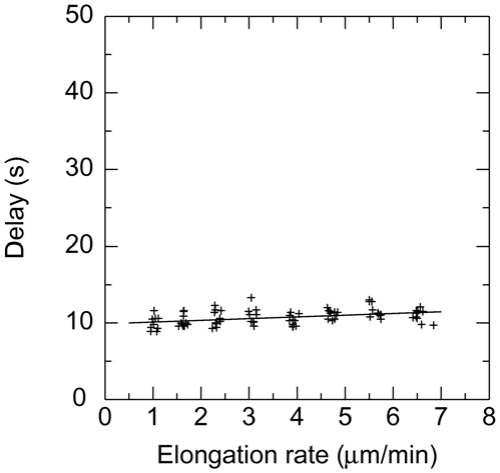
Catastrophe delay. Delay (s) before catastrophe as a function of the growing rate of microtubule (*µ*m/s). To compare with Figure 3.a in [10] for + end with 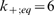, 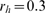, 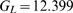, 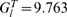, 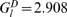,

**Figure 6:**
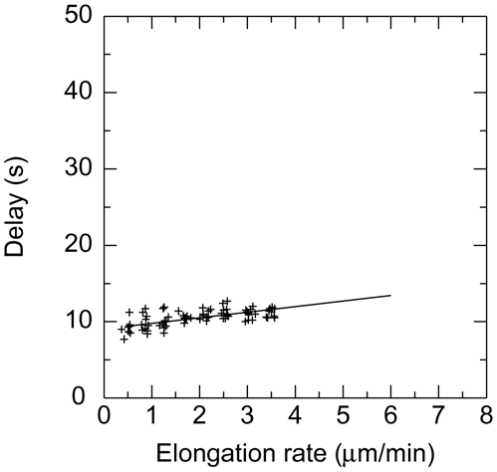
Catastrophe delay. Delay (s) before catastrophe as a function of the growing rate of microtubule (*µ*m/s). To compare with Figure 3.a in [10] for − end with 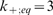, 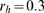, 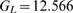, 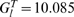, 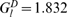.

To determine the best values of the parameters, we have calculated the values of 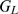, 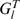 and 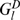 that reproduce the experimental data of Walker et al. taking 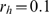, 0.2 and 0.3 and varying 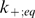 between 5 and 9 with an increment of 0.5 for the + end and between 2.5 and 5 with an increment of 0.25 for the − end. Note that for a given value of 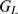, 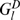 is simply determined by fitting the depolymerisation rate, 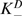, of a pure GDP-tubulin microtubule. We have first determined the best values of 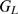, 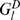 and 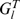 for various values of 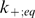 and 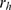 by comparing the growing rate and depolymerisation rate values of [14] for 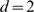 and 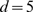. In Fig. 4, we present the values of the binding energies 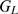, 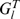 and 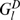 as a function of 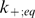 for the + and − ends, for 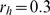. We have then compared the dynamics and the delay curves computed for these parameters with the experimental data. In particular, the hydrolysis rate 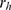 was determined by matching (Fig. 5 and 6) the experimental washout delay time of [10]. For this we simulated the dilution process of [10]: a 3 seconds delay followed by 5 seconds during which the free tubulin concentration decreases linearly. We have also chosen 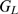 to be effectively the same for the − and the + ends and concluded that the best values of the required parameters are 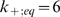, 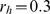, 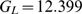, 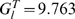, 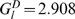 for the + end and 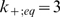, 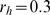, 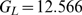, 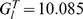, 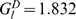 for the − end. We do not claim to have a good accuracy on these numbers. The error in 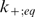 is probably of order 1 and this, by itself, induces errors in 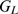, 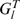, 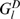 which are relatively large. Unfortunately with the experimental data available, it is not possible to determine 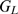 and 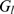 more accurately (see Fig. 3, 5 and 6).

Note that the individual dimers attachment rates 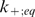 is not identical to the average microtubule growing rate 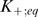. The difference comes from the interaction between neighbouring protofilaments which makes the microtubule dynamics non-linear.

**Figure 7:**
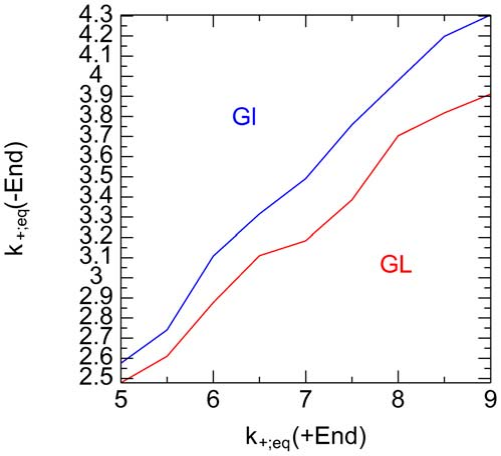
Association rates. Association rates 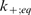 at the − end as a function of 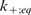 at the + end when both ends have the same binding energies 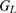 and 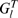.

Having computed 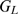 and 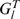 for various values of 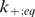 for the + and − ends of the microtubule we can read from Fig. 4 which values of 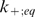 for the + and − end correspond to the same values of 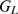 or 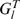. This is shown in Fig. 7 where we see that the two curves are close to each other, indicating that it is 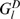 that differs between the 2 ends of the microtubule, while 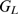 and 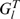 are effectively the same for both ends.

### Application of the model to microtubule structure

**Figure 8:**
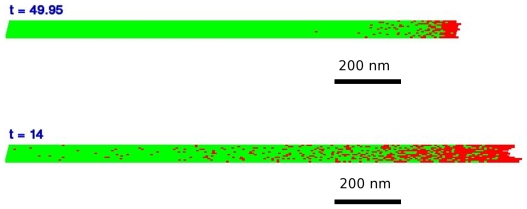
Typical Microtubule cap. red: GTP-tubulin, green GDP-tubulin. The microtubule is represented as unfolded on a plane for graphical convenience. Top) 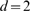, 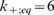, 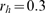, 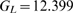, 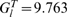, 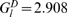. The microtubule sections shown are just under 200 dimers long. Bottom) Same as (b) for 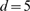.

In Fig. 8A and 8B we present two typical snapshots of the plus end of a growing microtubule (from movies S1 and S2 provided in the supplementary information) where the relative density, *d*, of free tubulin is different but all other parameters *i.e.* the binding energies and the dimer attachment rates are the same and as derived in the previous section. In Fig. 8A 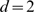 and in Fig. 8B 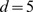. Moreover, the microtubules are represented in an unfolded configuration so one can see all 13 protofilaments with the top and the bottom protofilament forming the seam in the folded microtubule. These movies show that the microtubule cap length which is the distance from the tip to the last GTP tubulin dimer is proportional to the concentration of the free tubulin and in these simulations is *ca* 20nm in movie 1 (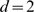) and *ca* 80 nm in movie 2 (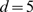).

**Figure 9:**
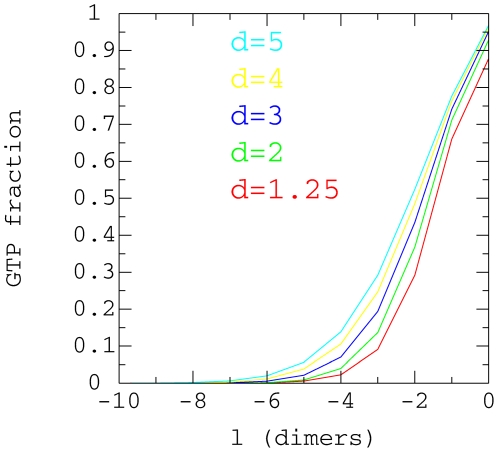
GTP-tubulin proportion in the cap crown. Average proportion of GTP-tubulin in the microtubule cap crown for 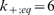, 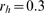, 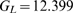, 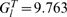, 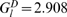 and for various free GTP-tubulin concentrations. The distance *l* is measured in dimer units from the bottom of the crown.

**Figure 10:**
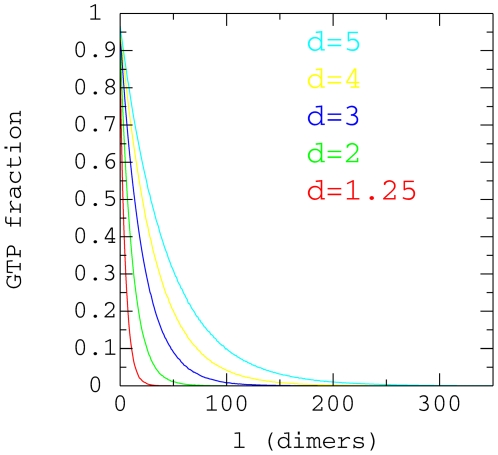
GTP-tubulin proportion in the cap core. Average proportion of GTP-tubulin in the microtubule cap core for 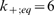, 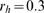, 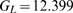, 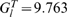, 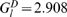 and for various free GTP-tubulin concentrations. The distance *l* is measured in dimer units from the bottom of the crown.

In order to analyse the size of the cap we performed a simulation where we sampled the distribution of GTP-tubulin dimer every second. We counted the average number of GTP tubulin dimers from a particular reference point over 100000 samples. This reference point was the base of the microtubule crown i.e. the point at which the 13 protofilaments at the tip are not folded into a complete cylinder (Fig. 2). Moreover, we counted in both directions: into the crown (Fig. 9) and into the core (Fig. 10) of the microtubule cap. Fig. 9 shows the average distribution of GTP-tubulin dimer in the crown for free tubulin concentrations between 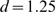 to 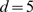 and for one protofilament. As the crown is almost exclusively GTP tubulin dimer, the graphs can be interpreted as representing the time distribution of the protofilament length in the crown and this generally fluctuates from 1 to 7 dimers but can be as high as 10 dimers. For example, we can interpret from the data in Fig. 9 that when 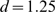, the crown is 2 dimers long only 30% of the time whilst for 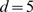 it is 2 dimers long 50% of the time.

**Figure 11:**
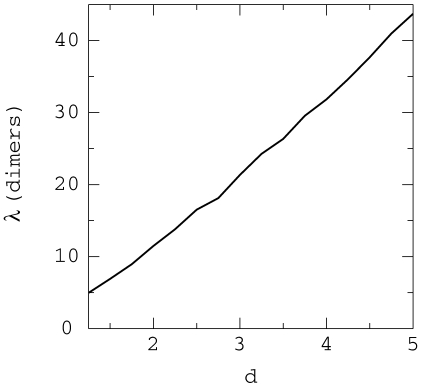
Cap core size. Size of the microtubule cap core as a function of the tubulin concentration obtained by fitting the GTP-tubulin concentration curve of figure (a) to an exponential 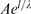 where *l* is the length measured in dimers. The size plotted is the value of *λ*.

When we measure the GTP-tubulin dimer in the core of the microtubule cap (Fig. 10), we see that the distribution of GTP tubulin dimer decreases exponentially with the distance (defined by *l*) from the reference point, the base of the crown. These data generated fit with the expression 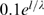 except near the crown base where the distribution of GTP tubulin dimer grows faster than an exponential. Therefore we can say that parameter *λ* is a good measure of the cap length (in tubulin dimer units). We have plotted the values of *λ* against the relative concentration of GTP tubulin dimer, *d*, in Fig. 11 showing that the cap size grows linearly with *d*.

It is interesting to note that if one had a specific marker for GTP-tubulin it might not be easy to measure directly the length of the GTP cap. Nevertheless, one could measure how the GTP-tubulin density decreases as one moves away from the tip of the microtubule. Fitting that density to an exponential curve, one would be able to measure the characteristic length *λ* and compare it to the prediction of our model shown in Fig. 11.

**Figure 12:**
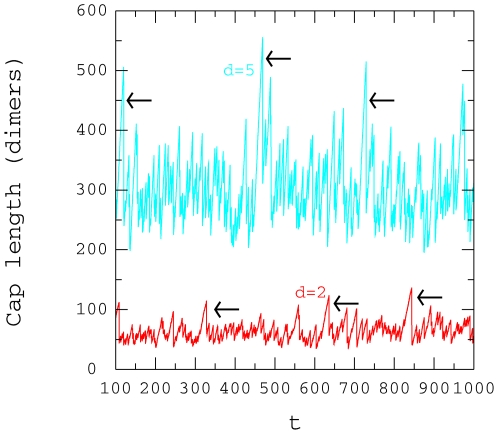
Cap length. Time variation of the microtubule cap length at the + end: 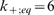, 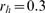, 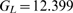, 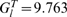, 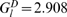. The arrows points to some sharp shortening.

**Figure 13:**
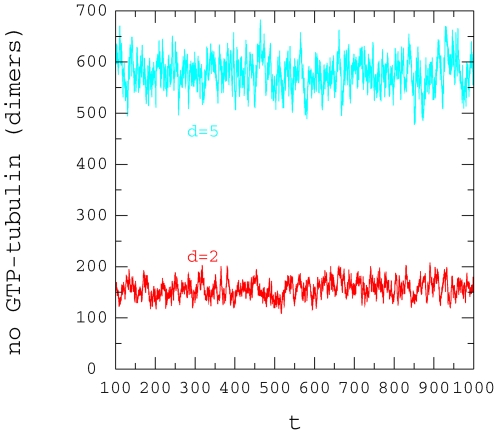
Number of GTP-tubulin dimers in the cap. Time variation of the microtubule size at the + end: 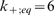, 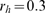, 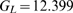, 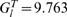, 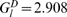.

**Figure 14:**
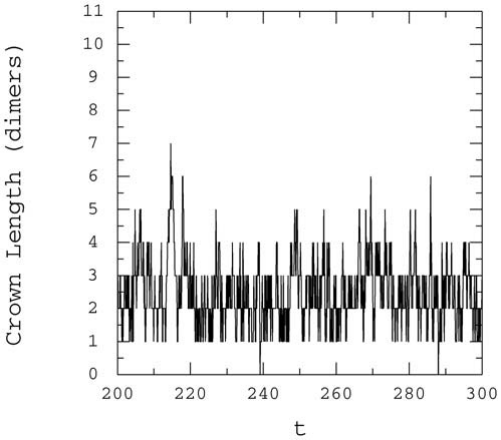
Cap crown length, 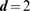. Time variation of the microtubule cap crown length at the + end: 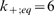, 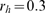, 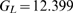, 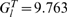, 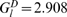.

Fluctuations in the length of the microtubule cap at the + end is evident over time as shown in Fig. 12 for two free tubulin dimer concentrations, 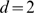 and 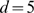. The sharp decreases in length occur when a single GTP-tubulin dimer takes a long time to hydrolyse to GDP dimer. For example, between the arrows in Fig. 12 the hydrolysis time is 30 s compared to the average GTP hydrolysis time of 3 s. The decreased length depicts the new length of the cap which is from the next closest GTP-tubulin dimer to the base of the crown. Moreover we can evaluate the length of the cap and this averages approximately 60 dimers for 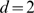 and 300 dimers for 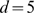. Another way of evaluating the size of the cap is to count the number of GTP-tubulin dimers over the 13 protofilaments. These data are shown in Fig. 13 and fluctuations in the number of GTP-tubulin dimers is apparent further supporting the variations in cap length seen in Fig. 14. Performing a similar analysis and considering only the crown, fluctuations in length occur from 1 to 7 GTP tubulin dimers when 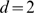.

It is also interesting to note that we have repeated these simulations where the number of protofilaments is 12 to 15 [1] and we see no change in the growing rate of the microtubule compared to the 13 protofilament microtubules used in our analysis. Moreover, when we change the monomer shift from the normal 3 tubulin monomers to 0-5 monomers at the seam, which occurs between protofilament 1 and 13, we again see no change in microtubule growing rates. These data indicate that the protofilament number and the displacement at the seam do not contribute directly to microtubule dynamics.

## Discussion

Here we have generated a Monte Carlo model of the assembly/disassembly of microtubules where we considered the rates of attachment and detachment of the tubulin dimer at the + and − ends as well as the hydrolysis rate of the GTP to GDP tubulin dimer within the microtubule. Our starting premise is that microtubules are in an aqueous solution of polymerised and free tubulin dimers. We assume that this is a perfect solution in a thermodynamic context. At the critical concentration of free tubulin dimer for assembly the system is at thermodynamic equilibrium. At this equilibrium we use the thermodynamic laws [12] to relate the ratio between the attachment and detachment rate of a GTP tubulin dimer to its binding energy to the microtubule Eq. 8 .

In comparing the development of our model with that of Van Buren et al 2002, we take into account the contribution of water to the entropy of the system ( Eq. 8 ). We also take the association rate 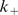 as a variable parameter rather than setting it to the average growing rate of microtubules. Furthermore, we have not set the GTP hydrolysis rate to zero at any point.

Our model is similar to the current model of Van Buren et al [9] with the following refinements. Firstly, we take into account the contribution of water to the entropy of the system which leads to the factor 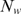 appearing in Eq. 8 and 11 . By treating the system as a solution, rather than a gas as in [9], we find that the longitudinal binding energies, 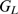 and 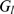, are systematically 4 kT smaller than in [9]. Secondly, in our hands (B.P. and K.P. independently) the calculations for 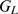 and 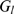 in [9] can only be valid if 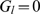. This cannot be the case otherwise the protofilaments in the microtubules would fall apart. One solution to this problem is to take 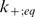 as a separate parameter that differs from the average microtubule protoflament growing rate 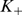 ( Eq. 16 ). However, this means that this value has to be determined from the experimental data and here we have done this. Thirdly, we have determined the values of 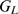 and 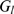 using a non-zero hydrolysis rate throughout microtubule polymerisation which was determined as 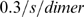 from the dilution experiments in [10]. In [9] the hydolysis rate was set to zero to determine 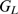 and 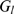 and then set to 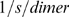 for their simulations. However, if the 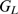 and 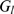 values are not adjusted when the hydrolysis rate is changed then the polymerisation rate of the microtubule will be less than the experimental value. Fourthly, for equation Eq. 12 to be valid the hydolysis rate has to be much smaller than the smallest value of 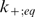 considered in [9] i.e. 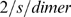 as explained in the derivation of equation Eq. 12 here in this paper.

Using our model we established that the dimer attachment rate is larger ( *ca.* 2 times) than the average microtubule growing rate which indicates that the microtubule dynamics is a complex stochastic process which depends crucially on the various configurations that the microtubule cap assumes ( *i.e* the shape, size and dimer constitution of the cap). We have fit all the parameters in our model to the experimental data of Walker et al [10], [14]. The parameters are, the GTP tubulin dimer attachment rate at both ends, the lateral binding energies of GTP and GDP tubulin dimers at both ends and the longitudinal binding energy, which we assume to be the same at the + and the − ends, which were all determined from the microtubule polymerisation experiments detailed in Walker et al [14]. In addition, the hydrolysis rate was determined from the microtubule polymerisation dilution experiment described in Walker et al [10].

Once the model had been fit to the experimental data we examined the structure of the microtubule cap. We noted from the length distribution of GTP tubulin dimers and the differences in the hydrolysis time of GTP that the GTP tubulin dimer cap is long and can be up to several microns in length dependent on the free dimer concentration (d) used in the simulation exercise. In the microtubule polymerisation experiments performed by Walker et al, they determined the growing rate of individual microtubules over a period of 6–8 s. They replaced the free tubulin dimer solution with buffer and measured the time it took for the microtubules to start to depolymerise, the time delay value. They found that the time-delay increased slightly with the growing rate of the microtubule and concluded that the GTP cap was only one dimer long. However, using our model to simulate their data we can reproduce their graphical data almost exactly including the scattering around the linear fit (cf Fig. 5 with Fig. 3 in [10]). This indicates that their data are compatible with a long microtubule cap and that their fluctuations (the observed scatter) is due to the stochastic nature of the polymerisation process rather than experimental errors.

Finally, we have found that neither the number of protofilaments nor the topology of the microtubule seam have any significant effect on the microtubule dynamics. This is important given the natural variation in protofilament numbers in different biological systems.

While we have compared the predictions of our model with the *in vitro* experimental data, it is important to point out that the model also applies to *in vivo* microtubles but only after the parameters of the model have been modified to reflect the differences between the buffer used in *in-vitro* experiments and the cytoplasm of a cell. A buffer and the cytoplasm differ by the type of solutes and their concentrations and these differences affect the thermodynamics properties of the system. A variation in the ion concentrations is likely to affect the binding energies of the tubulin dimers, though probably not by very much, and the variation of the entropy 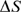, if the solution is not perfect. So the values of 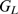 and 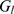 would have to be altered. The hydrolysis rate 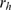 is also likely to differ and as ions are likely to interfere with the binding of the free tubulin dimers, the attachment rate 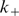 is likely to differ also. One must also bear in mind that different cells can have different tubulin isotypes and that this can also affect the values of the parameters of the model. So before one can apply our model to the *in vivo* situation all the model parameters would have to be fitted to the specific cell type.

## Supporting Information

Movie S1 Polymerisation of a 13 protofilament microtubule. d = 2, k+;eq = 6, rh = 0.1, GL = 12.4616, GlT = 9.50429, GlD = 2.84232.
(4.07 MB MPG)

Movie S2 Polymerisation of a 13 protofilament microtubule. d = 5, k+;eq = 6, rh = 0.1, GL = 12.4616, GlT = 9.50429, GlD = 2.84232.
(5.06 MB MPG)
